# Mental health over the life course: Evidence for a U‐shape?

**DOI:** 10.1002/hec.4614

**Published:** 2022-10-13

**Authors:** Hermien Dijk, Jochen Mierau

**Affiliations:** ^1^ Faculty of Economics and Business University of Groningen Groningen The Netherlands; ^2^ Accare Groningen The Netherlands; ^3^ Lifelines Cohort Study and Biobank Roden The Netherlands; ^4^ Team Strategy & External Relations University Medical Center Groningen Groningen The Netherlands

**Keywords:** age‐period‐cohort, mental health

## Abstract

Mental health problems impose substantial individual and societal costs over the life‐cycle. The age‐profile of mental health problems is, however, not well understood. Hence, we study the age‐profile of mental health while introducing minimal bias to reach identification. Using mental health data from the United States Panel Study of Income Dynamics we apply first difference estimation to derive an unbiased estimate of the second derivative of the age effect as well as an estimate up to a linear period trend of the first derivative. Next, we use a battery of estimators with varying restrictions to approximate the first derivative. Our results suggest that the age profile of mental health in the US is not U‐shaped and we find tentative evidence that the age‐profile could follow an inverse U‐shape where individuals experience a mental health high during their life course. Further analyses, using German and Dutch data, confirm that these results do not only apply to the US, but also to Germany and the Netherlands.

## 1 INTRODUCTION

Mental health problems are highly prevalent: approximately 20% of the working age population suffers from a mental disorder at any point in time and lifetime prevalence is estimated to be up to 50% [Organisation of Economic Co‐operation and Development (OECD), 2012]. This high prevalence results both in an extremely large burden of disease (Murray et al., 2012; Whiteford et al., 2013), as well as significant societal costs: mental health problems are estimated to be the leading cause of years lived with disability worldwide (Whiteford et al., 2013) and the societal cost of mental disorders is estimated to be 3%–4% of gross domestic product in OECD countries (OECD, 2012, 2014).

Over the last few decades, studies have indicated the importance of mental health for individuals' educational achievement and productivity (Currie, 2009; Currie & Stabile, 2006; Fletcher, 2010, 2014; Mousteri et al., 2019). In this light, it is important to understand the age‐pattern of mental health. If mental health negatively affects educational attainment and productivity, but is more prevalent or severe in middle‐aged or older individuals, the overall negative human capital effects of poor mental health might nevertheless be relatively small. However, if young individuals are more likely to suffer from poor mental health, the negative consequences of poor mental health might affect individuals' entire life course through its negative effects on human capital accumulation.

Importantly, potential interventions aimed at ameliorating the individual and societal costs of mental health problems could be targeted at various life‐cycle periods. Indeed, age seems to play a particularly important role in mental health problems and a host of studies have investigated the age‐pattern of mental health (Bell, 2014; Blanchflower & Oswald, 2008, 2016; Kino et al., 2019; Lang et al., 2011; Le Bon & Le Bon, 2014; Okui, 2020; Page et al., 2013; Park et al., 2018). The majority of these studies have found that mental health follows a U‐shaped pattern in age: young and old individuals generally experience better levels of mental health than individuals in, or close to, middle age (Blanchflower & Oswald, 2008, 2016; Lang et al., 2011; Le Bon & Le Bon, 2014; Okui, 2020). These results suggest that society would do well to invest more resources targeted at care and prevention of mental health problems among the middle aged.

Of the studies reporting U‐Shapes, a large share (Blanchflower & Oswald, 2016; Lang et al., 2011; Le Bon & Le Bon, 2014) use cross‐sectional evidence. However, investigating mental health trajectories over the life course is not without its caveats. By definition age, period and cohort are perfectly collinear (once an individual's age and the current year are known, it is possible to determine which year they were born). As a result, cross‐sectional evidence regarding which age groups currently experience lower or higher mental health provides insufficient knowledge on the age‐pattern of mental health, as it provides no evidence on whether the observed differences between age groups can be attributed to age or cohort effects. Hence, cross‐sectional evidence cannot be generalized to future cohorts and age groups, and can provide no indication of whether interventions should either be targeted at specific cohorts or specific age groups.

The distinction between cohort and age effects is important, because it specifically indicates which populations should be targeted with prevention policies. If the midlife dip is, in fact, due to a cohort effect, then preventive interventions are best targeted at other cohorts with similar risk‐factors for low mental health. Conversely, if the midlife dip in mental health is due to an age effect, preventive interventions, specifically, should be targeted at age‐groups slightly younger than midlife. Similarly, it is relevant to distinguish between age and period effects as they result in different predictions of future mental health. For example, if the period trend implies decreasing mental health over time, population mental health is likely declining over time, probably as a result of broader societal processes. If such a declining trend is the result of an age effect, as opposed to a period effect, then population mental health is not necessarily declining over time.^1^ Both scenarios warrant different policy responses and interventions.

Because of the fundamental collinearity between age, period and cohort effects, statistical analysis requires assumptions with various degrees of arbitrariness that cannot be tested. A number of approaches has been suggested to tackle this problem, especially in the related literature on the age effects of well‐being and life satisfaction, all with different assumptions and (dis)advantages. Importantly, in spite of how related mental health, well‐being and life satisfaction may be, for the purpose of this paper we refer to contributions regarding well‐being and life‐satisfaction mainly in the light of their methodological merits. Be that as it may, the various approaches suggested to tackle the Age, Period and Cohort (APC) problem often lead to conflicting results. For example, studies assuming cohort effects are negligible generally report U‐shapes in mental health, life satisfaction or well‐being (Blanchflower & Oswald, 2016, 2017; Graham & Pozuelo, 2017; Laaksonen, 2018; Lang et al., 2011; Le Bon & Le Bon, 2014), whereas studies using Hierarchical Age‐Period‐Cohort (HAPC) models consistently report no U‐shapes (Bartram, 2020; Beja, 2017; Bell, 2014; Yang, 2008). Since the nature of the APC problem prevents formal testing of many of these assumptions the true age‐profile remains unknown.

An alternative approach, proposed by De Ree and Alessie (2011) and Van Landeghem (2012) and used by Cheng et al. (2017)^2^ and Van Ours (2021), stands out because of its lack of need for arbitrary assumptions. By focusing on the first differences of life‐satisfaction or well‐being,^3^ these studies can identify age effects up to a linear trend. Hence, while the methods employed in these studies cannot prove the existence of a U‐shape in mental health as individuals age (since the linear trend remains unknown), they can provide proof when the U‐shape is nonexistent.

Nevertheless, the inability of these studies to identify the linear age‐trend is troubling, as this means that the true age‐profile still remains unknown. Therefore, we apply the method proposed by De Ree and Alessie (2011) and Van Landeghem (2012), but provide more information on the linear age‐effect by also displaying the results of a battery of estimations using varying cohort restrictions. That is to say, we use the least restrictive assumption on cohort effects by birth year available, that still allows for identification of the linear age‐effect, by assuming that only two out of all cohorts have equal cohort effects. We then vary this restriction by choosing two different cohorts in each new estimation, resulting in a multitude of estimates.

To this end, we use data from three countries: the US Panel Study of Income Dynamics (PSID), as well as the German Socio‐Economic Panel (SOEP) and the Dutch Longitudinal Internet Studies for the Social sciences (LISS) panel. Our results indicate that the U‐shape is not the dominant functional form in the relationship between mental health and age. In contrast, we find that the age‐related profile of mental health could potentially follow an inverse U‐shape, suggesting that young individuals and older adults might be particularly at risk of developing mental health problems.

Aside from its relevance to effective targeting of mental health policy toward relevant risk groups, the methodological contribution of this paper to the literature is threefold. Firstly, to our knowledge, this paper is the first to apply the first difference approach proposed by De Ree and Alessie (2011) and Van Landeghem (2012) to mental health for a wide range of ages, including both relatively young and old individuals. As such, this study is the first to investigate the life‐cycle pattern of mental health with substantially less restrictive assumptions than earlier contributions. Secondly, this paper introduces a relatively new approach regarding cohort restrictions. While it is not new to restrict a single cohort when using age, period and cohort effects as control variables, the current adaptation of the method—restricting only a single cohort when age effects are of primary interest and varying this cohort restriction across estimations—is new. Lastly, in contrast with a large portion of the literature, this paper presents results from multiple estimation strategies for the linear age effect. This is important, as different methodological choices appear to lead to different outcomes.

This paper is structured as follows. Section 2 discusses the existing literature on the age‐profile of mental health and well‐being as well as the commonly applied methodologies. Section 3 provides a detailed explanation of the econometric methods used in this paper, after which Section 4 describes the data used for the main analysis. Results are provided in Section 5 and Section 6 contains the further analyses. Section 7 provides a detailed discussion of the main result, after which Section 8 provides a conclusion.

## 2 BACKGROUND

Before delving into the literature, it is important to understand the APC problem and the proposed solutions to the problem. A much quoted (Bell & Jones, 2013, 2014, 2015) example of the distinction between age, period and cohort effects is the conversation between a senior worker (A) and a junior worker (B) from Suzuki (2012):

A I can't seem to shake off this tired feeling. Guess I'm just getting old. [Age effect]

B Do you think it's stress? Business is down this year, and you've let your fatigue build up. [Period effect]

A Maybe. What about you?

B Actually, I'm exhausted too! My body feels really heavy.

A You're kidding. You're still young. I could work all day long when I was your age.

B Oh, really?

A Yeah, young people these days are quick to whine. We were not like that. [Cohort effect] (Suzuki, 2012).

The problem is that every combination of two items of this list perfectly predicts the third. In other words, age, period and cohort effects together exhibit perfect collinearity. As a result, they cannot be estimated in a standard regression equation. Many methodological solutions have been proposed to solve this problem, each with its own caveats.

We will not only report literature regarding the age‐profile of mental health, but also the age‐profile of life‐satisfaction and well‐being. These three concepts are closely related, as individuals with low life‐satisfaction or well‐being are more likely to report lower mental health and vice versa and, as such, these strands of literature have been intertwined (Galambos et al., 2020). Nevertheless, as mentioned above, for our purpose we refer to the well‐being and life‐satisfaction literature mainly with regard to their methodological contributions.

Perhaps the simplest method to circumvent the APC problem is by simply assuming that either period, or cohort effects are negligible and can thus be ignored. Multiple studies have assumed that cohort effects are irrelevant (Bittmann, 2020; Blanchflower, 2021; Blanchflower & Oswald, 2016, 2017, 2019; Graham & Pozuelo, 2017; Laaksonen, 2018; Lang et al., 2011; Le Bon & Le Bon, 2014). Others have instead assumed that period effects are irrelevant, which makes it possible to estimate age effects using fixed effects approaches (FitzRoy et al., 2014; Frijters & Beatton, 2012; Kageyama & Sato, 2021; Kassenboehmer & Haisken‐DeNew, 2012; Piper, 2015).

Another method, which requires slightly weaker assumptions, is to assume that age, period and cohort effects are all relevant, but that either age groups, periods or cohorts close together have equal coefficients. For example, using this assumption, one can estimate a regression using single‐year age dummies, single‐year period dummies, but 5‐ or 10‐year cohort dummies. Some studies have followed this approach (Blanchflower & Oswald, 2008; Easterlin, 2006; Lin et al., 2015; Page et al., 2013).

Others have used Hierarchical APC (HAPC) models, which require the assumption that cohort and period effects are completely random (Bartram, 2020; Beja, 2017; Bell, 2014; Yang, 2008).

The use of parameter restrictions has often been criticized (Bell & Jones, 2013, 2014; De Ree & Alessie, 2011; Luo & Hodges, 2016; O'Brien, 2017). By relying on arbitrary assumptions to reach identification, all of the studies above are likely to suffer from biases of unknown size. For example, by assuming cohort effects are negligible, estimated age‐effects include cohort effects as well as age effects.

To circumvent this issue, several authors have attempted to identify age effects using only minimal assumptions by focusing on the second derivative of the age profile (Cheng et al., 2017; De Ree & Alessie, 2011; Van Landeghem, 2012; Van Ours, 2021). The analyses in these studies can identify age patterns up to a linear trend; they can identify the second derivative of the age profile, but not the first.

This means that the true age‐pattern remains unknown. For example, the second derivative of age might indicate the existence of a U‐shape, but if the first derivative is sufficiently large, mental health, life satisfaction, or well‐being is nevertheless continuously increasing over the life course.^4^ In such a case, a statistically significant second derivative only means that there is significant curvature in the upward sloping age‐trend. Hence, while the methods employed in these studies cannot prove the existence of a U‐shape in mental health as individuals age (since the linear trend remains unknown), they can prove that the U‐shape is nonexistent.

Some similar methods are presented in Kino et al. (2019), Park et al. (2018), and Okui (2020), relying on assumptions around the linear period or cohort trend.

Of the studies cited here, 10 focus on mental health (Bell, 2014; Blanchflower & Oswald, 2008, 2016; Kino et al., 2019; Lang et al., 2011; Le Bon & Le Bon, 2014; Okui, 2020; Page et al., 2013; Park et al., 2018; Van Ours, 2021). Five of the 10 papers cited here do find a U‐shaped age profile of mental health variables (Blanchflower & Oswald, 2008, 2016; Lang et al., 2011; Le Bon & Le Bon, 2014; Okui, 2020). Three studies (Kino et al., 2019; Page et al., 2013; Park et al., 2018) provide no clear evidence in favor of or against the existence of a U‐shape. However, these studies only investigate the rather extreme case of suicide and are not necessarily focused on the existence of a U‐shape.

Only two studies cited here explicitly report no U‐shaped age profile in mental health (Bell, 2014; Van Ours, 2021). Bell (2014) argues that previous findings of a U‐shape are the result from confounding of cohort effects and that, instead, mental health declines as individuals age.

This overview of the literature shows that there is no panacea when it comes to APC estimation. Any method for identifying APC effects either has a large probability of misspecification (parameter restrictions) or is underidentified (only inferring information about the second derivative). As a result, different methods can lead to different outcomes. This is clearly illustrated when studies assuming cohort effects are negligible (Bittmann, 2020; Blanchflower, 2021; Blanchflower & Oswald, 2016, 2017, 2019; Graham & Pozuelo, 2017; Laaksonen, 2018; Lang et al., 2011; Le Bon & Le Bon, 2014) are compared to studies HAPC models (Bartram, 2020; Beja, 2017; Bell, 2014; Yang, 2008). The first category of studies consistently reported U‐shapes for mental health, life satisfaction or well‐being, while the latter consistently reported no U‐shapes. As a result, there is no consensus on the exact age‐profile of mental health, life satisfaction and well‐being.

In the sections that follow we investigate the age‐profile of mental health using US panel data. To ensure that our results are not the artifacts of methodological choices, we will first estimate the second derivative using first differences, after which we will approximate the first derivative using a battery of parameter restrictions. Both methods will be explained in more detail below.

## 3 METHOD

### 3.1 Second derivative

We base our analysis on Van Landeghem (2012) and De Ree and Alessie (2011) who both show that identification of the second derivative can be obtained by taking first differences from the dependent variable and regressing them on age as well as a set of year dummies.^5^ That is, assume our Data Generating Process (DGP) is given by:

(1) $$MH_{i,t}=\beta_{0}+\beta_{1}age_{i,t}+\beta_{2}age_{i,t}^{2}+\tau year_{t}+\gamma_{t}+\sum_{\phi =1905}^{C}\alpha_{\phi }cohort_{i}\left(\phi \right)+\epsilon_{i,t},$$

where *MH*_*i*,*t*_ denotes the mental health score of individual *i* in year *t*, *age*_*i*,*t*_ denotes the age of individuals *i* in year *t*, *τ* is the parameter denoting a linear period‐effect and *γ*_*t*_ denotes the deviation from the linear period‐effect, *cohort*_*i*_(*ϕ*) denotes a set of cohort dummies taking value one if *ϕ* equals the birth year of individual *i* and zero otherwise and *ϵ*_*i*,*t*_ denotes the error term. The parameters *β*_1_ and *β*_2_ are the parameters of interest, since they determine whether mental health is U‐shaped over the life‐course. By first differencing Equation (1), and using the fact that $age_{i,t}^{2}-age_{i,t-1}^{2}=2age_{i,t}-1$,^6^ this can be rewritten as:

(2) $$\Delta MH_{i,t}=\left(\beta_{1}-\beta_{2}+\tau \right)+\left(\gamma_{t}-\gamma_{t-1}\right)+2\beta_{2}age_{i,t}+{\tilde{\epsilon }}_{i,t},$$

where ${\tilde{\epsilon }}_{i,t}=\epsilon_{i,t}-\epsilon_{i,t-1}$. Hence, we can identify *β*_2_ by using first differences of the mental health scores as dependent variables in a regression analysis with age multiplied by two and a set of year dummies as independent variables. We can only identify *β*_1_ with this method if we are willing to make an assumption about the linear period trend *τ*.

Note that in the case of biennial data we can rewrite Equation (1) into

(3) $$\frac{1}{2}\bar{\Delta MH_{i,t}}=\left(\beta_{1}-2\beta_{2}+\tau \right)+\frac{1}{2}\left(\gamma_{t}-\gamma_{t-2}\right)+2\beta_{2}age_{i,t}+{\bar{\epsilon }}_{i,t},$$

where ${\bar{\epsilon }}_{i,t}=\frac{1}{2}\left(\epsilon_{i,t}-\epsilon_{i,t-2}\right)$ and $\bar{\Delta MH_{i,t}}=MH_{i,t}-MH_{i,t-2}$, using the same trick as was used for Equation (2) and multiplying both sides by $\frac{1}{2}$ to make estimation results from Equation (3) more easy to compare to those of Equation (2). Since we use both annual and biennial data in this paper, we will estimate Equation (3) for biennial data and Equation (2) for annual data.

#### 3.1.1 Another second derivative approach

Cheng et al. (2017) propose a variation on the second derivative approach in this section. They estimate the age‐effect of life satisfaction in two stages. In the first stage they detrend the first differences of life satisfaction by regressing it on a set of time dummies. From this equation they estimate the residuals, which are then used as the dependent variable in a regression with a constant and a linear age‐variable (and possible control variables). This way they hope to identify the equivalent of *β*_1_ and *β*_2_ in Equations (1) and (2).

However, this approach also cannot disentangle the linear period‐effect from the linear age‐effect, and hence it cannot identify *β*_1_. Moreover, in case the first stage contains a full set of year dummies,^7^ the second stage will regress a centered variable (the residuals from the first stage) on an uncentered variable (age). Since the centered variable will have a mean equal to zero, the estimated constant in the second stage will not be an approximation of *β*_1_, but instead it will have a value such that ${\hat{\lambda }}_{1}+{\hat{\lambda }}_{2}{\bar{age}}_{i,t}=0$, where ${\hat{\lambda }}_{1}$ would be the estimated constant in the second stage, ${\hat{\lambda }}_{2}$ would be the estimated coefficient for age in the second stage and ${\bar{age}}_{i,t}$ would be the mean age of the sample used for estimation. If one were to misinterpret ${\hat{\lambda }}_{1}$ and ${\hat{\lambda }}_{2}$ as approximations of *β*_1_ and *β*_2_, respectively, one would wrongly conclude that individuals experience a mental health low, or mental health high, at $-{\hat{\lambda }}_{1}/{\hat{\lambda }}_{2}$, which is equal to the mean age of the sample used for estimation.^8^ Consequently, since this approach would lead to misleading results regarding *β*_1_, we will not apply it in this paper.

### 3.2 First derivative

We will use parameter restrictions on cohorts to approximate the first derivative with respect to age. The least restrictive parameter restrictions model that allows for both linear age‐ and year‐effects as well as non‐linear cohort‐effects, but is still identified, is one where only two out of all cohorts are expected to have equal coefficients. This model is still biased, but the bias is minimized to the difference between the two cohort effects that are assumed to be equal. Additionally, if the average yearly change in cohort effect approximates zero, on average our estimation of the linear age‐effect should be close to the true coefficient.

Therefore, we estimate models of mental health using Ordinary Least Squares (OLS) regression with cluster robust standard errors with a continuous age variable, age squared, a set of year dummies and a set of (restricted) cohort dummies as independent variables. To reach identification the period dummies start from the second available year and the cohort dummies consist of a full set of cohort dummies minus a reference cohort and with the restriction that one cohort has a coefficient equal to the cohort from the previous birth year.^9^

## 4 DATA

We use data from the PSID for the main analysis. The PSID started in 1968 and consists of a nationally representative sample of individuals living in families. From 2001 onwards, with the exception of 2005, the family interviews contained six questions belonging the abbreviated Kessler psychological distress scale (K‐6) which were asked to the head and/or the partner of head of the household. The K‐6 is a broad screener that can be used to assess non‐specific psychological distress and the prevalence of serious mental illness in the general population (Kessler et al., 2002, 2010) and has been well validated (Furukawa et al., 2008; Furukawa et al., 2003; Gill et al., 2007; Prochaska et al., 2012). We will use this variable as a measure of mental health. The family interviews were held biennially.

The K‐6 consists of six questions asking how often individuals felt certain negative emotions during the last 30 days (sadness, nervous, restless, hopeless, that everything was an effort, worthless) which they can answer on a scale from 1 (all of the time) to 5 (none of the time) (Kessler et al., 2002). To create the overall K‐6 score, the scores from the different questions are summed into a single score. For convenience, we linearly transformed this single score so that it ranges from 0 to 100, where 0 indicates that an individual answered “all of the time” to all six questions and a score of 100 indicates that an individuals answered “none of the time” to all six questions.^10^

In our sample for analysis we included only the direct respondents of the family interview, as they were the ones answering the K‐6 questions. Additionally, we defined age as an individual's age on December 31 of the relevant survey year. Some individuals indicated a combination of age and birth year that is impossible given the survey year. These observations from 127 individuals were excluded from our sample. Summary statistics of our final sample can be found in Table 1.^11^ Our first difference approach requires the presence of at least two consecutive observations of the K‐6 for each individual in our set. As a result we lose 3127 out of 14,251 individuals. However, there is almost no variation between both samples in terms of age, year of birth and K‐6 score. Detailed summary statistics of this second sample can be found in Table A3 in Appendix A1. Hence, we do not expect this to influence our results.

**TABLE 1:** Population summary statistics

	*N*	Mean	Standard deviation	Minimum	Maximum
Age	14,251	38.96	15.81	16	99
Year of birth	14,251	1966.10	17.92	1902	1997
Year	14,251	2005.06	4.91	2001	2015
Gender					
Female	7856	55.13%			
Male	6395	44.87%			
K‐6 score (0–100)	14,251	83.93	17.27	0	100

*Note*: Summary statistic at first observation (baseline) for each individual.

**FIGURE 1:**
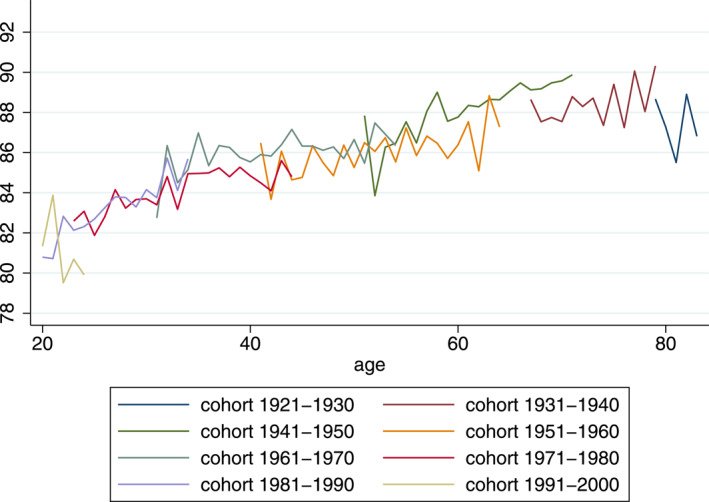
Average scores for: K6 (0–100 scale)

**FIGURE 2:**
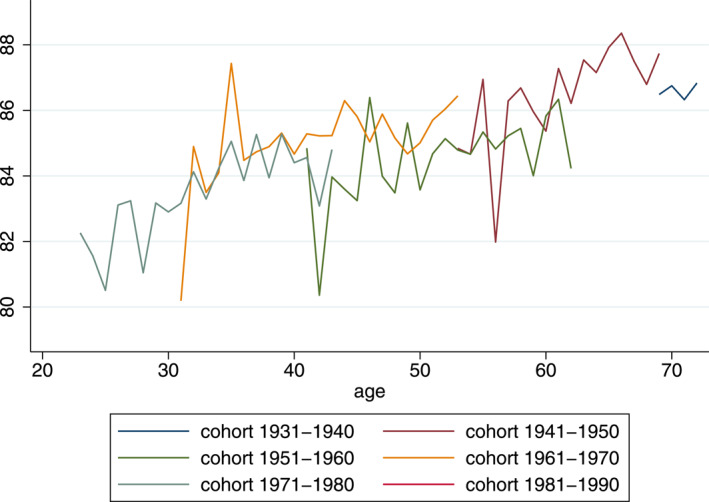
Scores women: K6 (0–100 scale)

**FIGURE 3:**
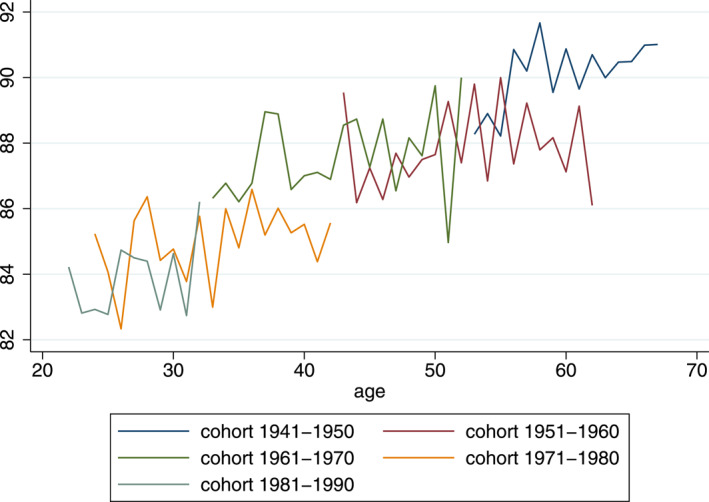
Scores men: K6 (0–100 scale)

Figures 1, 2, 3 provide a graphical analysis of the relationship between mental health and age, period and cohort. In the graph, all years are pooled together and age‐specific average K6 scores are stratified by 10‐year cohort.^12^ It is interesting to note that the mental health measure does not show a strong U‐pattern and that this is the case for both men and women. Mental health appears to increase at every age until individuals are around 70, after which—at least for women—it declines slightly.

Another interesting observation is that visible cohort‐ or period‐effects appear to be small: values for equal age groups of different cohorts are generally very similar. While this provides no conclusive evidence, it suggests that the patterns that we do see in the graphs might be due to age effects.

## 5 RESULTS

### 5.1 Second derivative

To ensure that our estimates are robust to autocorrelation we have used cluster robust standard errors. Additionally, to reduce any effects of over‐ or under‐sampling in the PSID we performed our estimations with PSID family sample weights. Table 2 provides the results of the estimation of Equation (3). It is interesting to note that our estimation results for *β*_2_ do not vary dramatically across genders, both have a statistically significant estimate of −0.004. These negative coefficients indicate that there is no U‐shape in mental health over the life course, as a U‐shape should have a positive second derivative.

**TABLE 2:** Estimation results: second derivative

$\frac{1}{2}\Delta$ K6 (0–100)	Full sample	Women	Men
2*age*_*i*,*t*_	−0.004***	−0.004***	−0.004***
	(0.001)	(0.001)	(0.002)
Constant	0.600***	0.759***	0.383
	(0.158)	(0.205)	(0.246)
Clustered SE	Yes	Yes	Yes
Observations	37,228	23,114	14,114
Number of ID	11,124	6733	4391

*Note*: Clustered standard errors in parentheses.

***p < 0.01, **p < 0.05, *p < 0.1.

On the contrary, the estimation results for the second derivative with respect to age (*β*_2_) indicate that there might be an inverse U‐shape in mental health over age. However, whether this inverse U‐shape truly exists depends on the linear term, which is not identified. If we are, however, willing to assume that there exists no linear time trend (*τ* = 0), women's mental health would be continuously increasing until they reach old age (they reach their peak mental health at age 85), whereas men would experience a more profound inverse U‐shape: given our current estimate, mental health would peak at age 44. Regardless of the size of the linear age‐effect, our finding of a negative second derivative disproves the existence of a U‐shaped age profile in mental health.

### 5.2 First derivative

**FIGURE 4:**
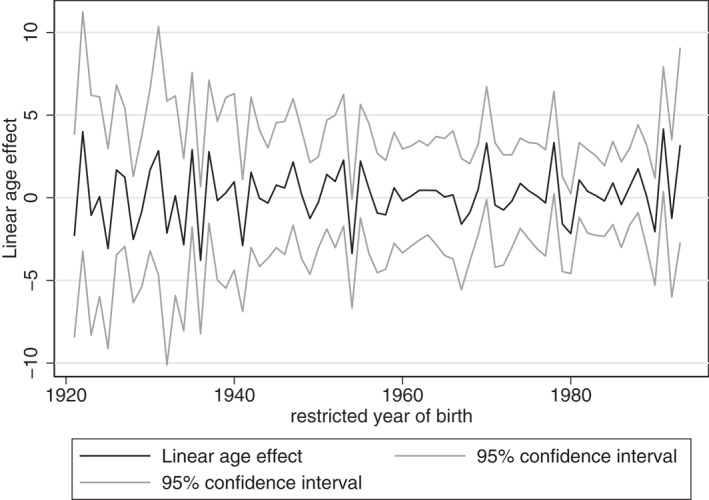
Estimates for linear age‐effect

**FIGURE 5:**
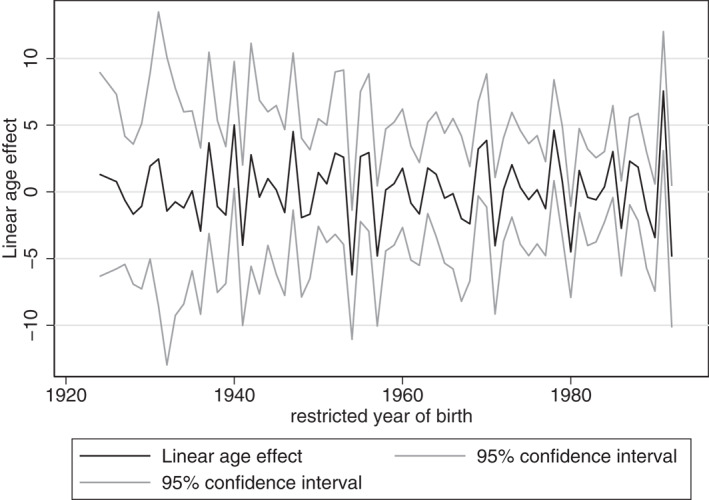
Linear age‐effect: women

**FIGURE 6:**
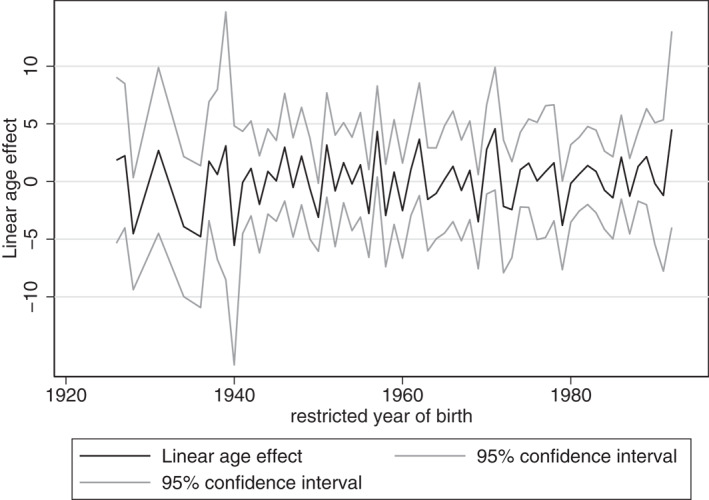
Linear age‐effect: men

Figures 4, 5, 6 provide the estimated coefficients for the linear age‐effect for different cohort restrictions.^13^ For each restricted year of birth, the coefficient of that cohort is restricted to be equal to the coefficient of the previous cohort. That is, if the restricted cohort is the one born in 1921, then the coefficient for the cohort effect of 1921 is assumed to be equal to that of 1920.^14^

For the overall sample, the parameter restrictions result in estimations of the linear age‐effect that vary between −3.777 and 4.155. Given these two extremes, mental health could peak at any age during the life course. Consequently, these estimates indicate that mental health could be U‐shaped over the life course, but they provide no proof, as mental health could also be continuously increasing or decreasing over age. This would mean that both young and older adults could be more at risk for mental health problems.

For women and men separately, we find similar results of linear age‐effects that vary between −6.211 and 7.555, and −5.538 and 4.579, respectively. Hence, according to these results, mental health could peak at any age during the life course, or be continuously increasing or decreasing over age.

## 6 FURTHER ANALYSIS

### 6.1 Functional form

It could be that the DGP in this study is too restrictive. It is relatively easy to test this hypothesis by assuming a less restrictive DGP. Following Van Landeghem (2012), we estimate Equation (3) using a set of dummies for age instead of a single continuous age variable. The age‐dependent second derivative is then given by differencing the coefficients of the age dummies. If the initial second order polynomial assumption is correct, the estimated age‐dependent second derivative should only exhibit minimal variation around a straight line. If instead the pattern of the age‐dependent second derivative is more complicated, this would suggest that our initial assumed DGP is incorrect.

**FIGURE A1:**
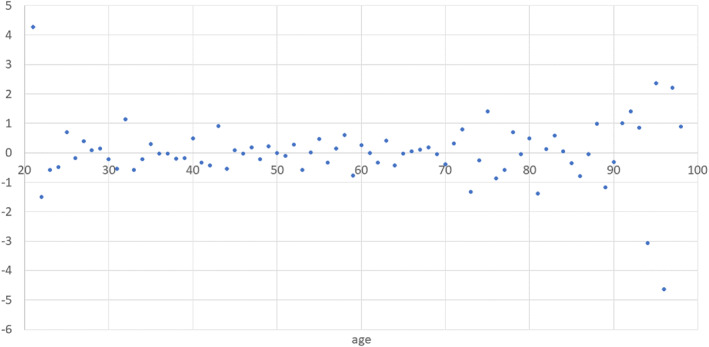
Age‐dependent second derivative

A plot of the age‐dependent second order derivatives is given in Figure A1 in Appendix A2. As the graph clearly shows, the age‐dependent derivative only shows minimal variation around a straight line. Additionally, a Ramsey RESET test (Ramsey, 1969) of Equation (2) to assess the null hypothesis for no misspecification provides *p*‐values of 0.039, 0.299 and 0.269 for the entire sample, women and men, respectively. While the small *p*‐value for the entire sample might suggest that the analysis suffers from some form of misspecification, the large *p*‐values of the separate specifications for men and women suggest that this might largely be due to gender differences. Hence, the choice of a second order polynomial for age in Equation (1) does not appear to cause misspecification. Consequently, the conclusions that the age‐profile of mental health is not U‐shaped and that it might even follow an inverse U‐shape appear to be valid.

### 6.2 Attrition

There might be a possibility that individuals of certain ages with certain mental health levels are more likely to drop out of the sample. If this is the case our estimate of the second derivative would be biased. When we perform the second derivative estimation including only those individuals that were present in all waves, we find an estimate for the second derivative that is slightly higher, but still negative and statistically significantly different from zero (*p* < 0.05) and not statistically significantly different from our previous estimations (*p* > 0.10) (see Table A4 in Appendix A3). Consequently, our results appear to be relatively unaffected by panel attrition.

### 6.3 Period and cohort trend

So far, we have provided two estimation strategies for the linear age‐effect. The first estimation strategy (using first differences) required the assumption of a negligible period trend in order to identify the age effect. The second strategy (using varying cohort restrictions) required the assumption of a negligible cohort trend. The degree of certainty about the first derivative hinges on the credibility of these assumptions. In other words, is there reason to believe either a linear period or cohort trend is more or less likely? In Appendix A6 we provide two graphs: Figure A3 provides average K‐6 (0–100) scores per year of observation and Figure A4 provides average K‐6 (0–100) scores per cohort. Note that both provide no conclusive evidence on the linear trend, as the averages are biased with age effects, and period or cohort effects. Nevertheless, average K‐6 (0–100) scores show strikingly little variation over time, suggesting that perhaps the assumption of no linear period trend might not be too unrealistic.

### 6.4 Control variables

Our main results indicate that there is no U‐shape in mental health over age. This is not in line with a large share of the literature, which frequently reports a U‐shape (Blanchflower & Oswald, 2008, 2016; Lang et al., 2011; Le Bon & Le Bon, 2014; Okui, 2020). However, one important difference between our study and a large share of the literature is the use of control variables other than cohort and period variables to estimate the age effects of mental health (e.g., Blanchflower & Oswald, 2008; Lang et al., 2011, and to some extent Blanchflower & Oswald, 2016).

This can lead to differences in results, since including additional control variables implies estimating the age‐pattern conditional on the controls remaining constant, for example, the age‐pattern in mental health for someone who earns a constant level of income throughout life. Therefore, through the use of control variables, Blanchflower and Oswald (2008) and Lang et al. (2011) measure something different (a residual age‐effect) from the crude age‐effect investigated in this study, which might lead to and account for different results, and thus different conclusions about the existence of a U‐shape.^15^

Consequently, we also perform our estimation of the second derivative using a set of (differenced)^16^ control variables on income decile, marital status, employment status, education level, state of residence, and whether individuals lived with children under 18. We find second derivatives of −0.003 for men, women and the full sample (*p* < 0.10, *p* < 0.10 and *p* < 0.05, respectively). Hence, even when these specific controls are included, we find a negative second derivative, indicating that the existence of a U‐shape is unlikely. Detailed results can be found in Table A5 in Appendix A4.

### 6.5 The Netherlands & Germany

Our finding that mental health is inversely U‐shaped over the life course runs counter to the current literature, which more often than not reports a U‐shape in mental health, well‐being or life satisfaction. It might be though that this is simply due to the fact that we focus on the US, which might be the exception to the U‐shaped rule (Blanchflower & Oswald, 2009). To test this assumption, we repeat our analysis of the second derivative on data from the Netherlands and Germany.

For our analysis of the Netherlands, we use data from the Dutch LISS panel,^17^ which consists of a representative sample of the Dutch population. The LISS panel contains data on individual mental health for the years 2007–2017, with the exception of 2014. Mental health is measured using the abbreviated five‐question version of the Mental Health Inventory (MHI‐5) (Ware & Sherbourne, 1992), which is a widely used and well validated instrument, specifically for mood and anxiety disorders (McCabe et al., 1996; Rumpf et al., 2001; Veit & Ware, 1983). The MHI‐5 can be summarized in a score ranging from 0 to 100, where higher scores indicate better mental health.

For Germany we use data from the SOEP. The SOEP is an ongoing long‐run household panel with data on German households starting from 1984 of which we use the data up to and including 2016. A large number of papers have used SOEP to investigate a U‐shape in life‐satisfaction and well‐being (e.g., Baetschmann, 2014; Cheng et al., 2017; De Ree & Alessie, 2011; FitzRoy et al., 2014; Frijters & Beatton, 2012; Kassenboehmer & Haisken‐DeNew, 2012; Rohrer et al., 2018; Van Landeghem, 2012), but, to our knowledge, currently no study has used SOEP to investigate the age‐profile of mental health.

For mental health we use the Mental Component Summary scale (MCS), which is computed from answers to the SF‐12v2 questionnaire using factor analysis (Andersen et al., 2007). The MCS is calibrated such that the population average is close to 50 and the standard deviation is close to 10, with higher scores implying better mental health. The MCS is available from 2002 onwards, hence we use data from all even years between 2002 and 2016, which resulted in data from eight different years.^18^

**FIGURE A2:**
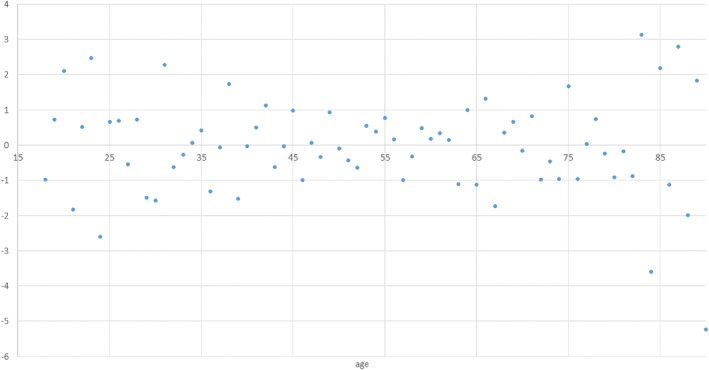
Age‐dependent second derivative: Netherlands. Second derivative estimates for individuals aged >90 are not shown due to extreme outliers as a result of limited sample sizes for these age groups

**FIGURE A3:**
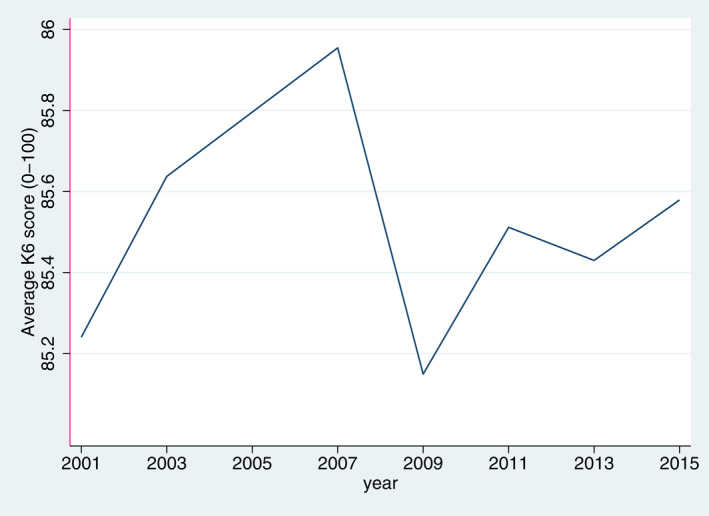
Average K6 scores per year (0–100)

**FIGURE A4:**
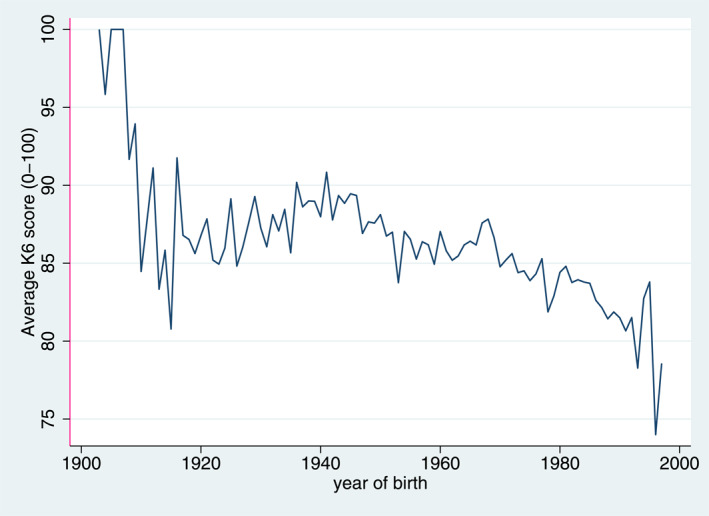
Average K6 scores per cohort (0–100)

Estimated second derivatives for both datasets can be found in Table A6 of Appendix A5. For Germany we find relatively similar results to the US, indicating that the non‐existence of the U‐shape in mental health in the US is not an isolated case. For the Netherlands we find a statistically insignificant second order derivative of 0.001 (SE: 0.002), indicating that either the second derivative is relatively close to 0 or our current assumed DGP might be misspecified for the Netherlands. We can test this last possibility in the same way as we did previously. Results for the age‐dependent second derivative for the Netherlands can be found in Figure A2 of Appendix A5. No clear age pattern emerges when looking at the age‐dependent second derivative for the Netherlands, suggesting that the second derivative might indeed be close to zero. Additionally, a Ramsey RESET test (Ramsey, 1969) could not reject the null hypothesis of no misspecification with a *p*‐value of 0.136. Consequently, the U‐shape in mental health is not only absent when using US data, but also when using German and Dutch data.

## 7 DISCUSSION

Our results consistently indicate that there is no U‐shape in mental health over the life‐cycle, and that this finding is not limited to just the US. While this finding is consistent with Bell (2014) who also finds negative second derivative, it contrasts with much of the literature, which frequently reports a U‐shape (Blanchflower & Oswald, 2008, 2016; Lang et al., 2011; Le Bon & Le Bon, 2014; Okui, 2020), which raises the question why our results differ.

One possible reason for the difference in results is that three of the five studies reporting U‐shapes in mental health over age (Blanchflower & Oswald, 2016; Le Bon & Le Bon, 2014; Okui, 2020) use some form of mental healthcare use as proxies for mental health. The underlying assumption is that if the age profile of mental healthcare use consists of an inverse U‐shape, the age profile of mental health must also be U‐shaped. However, mental healthcare use might not be an adequate proxy for mental health and the reliability of healthcare use as a proxy might be dependent on age (Alonso et al., 2007; Clement et al., 2015; Wang et al., 2005). This could bias the earlier results.

Additionally, three of the five studies do not control for cohort effects, assuming that these are zero (Blanchflower & Oswald, 2016; Lang et al., 2011; Le Bon & Le Bon, 2014). However, this might not be the case, leading to biased results. Even when cohort effects are taken into account (Blanchflower & Oswald, 2008), most of the studies finding a U‐shape in mental health (Blanchflower & Oswald, 2008, 2016; Lang et al., 2011; Le Bon & Le Bon, 2014) use superfluous parameter restrictions, suggesting that cohort effects are perhaps not adequately controlled for (Bell & Jones, 2013).^19^

In this study we have used a very narrow definition of mental health. The WHO (2014) has defined mental health as “a state of well‐being in which every individual realizes his or her own potential, can cope with the normal stresses of life, can work productively and fruitfully, and is able to make a contribution to her or his community.” While the questionnaires used in this study capture some of this definition of mental health, they certainly do not encompass all of the definition by the WHO (2014). Specifically, the K‐6 focuses more on the presence or absence of psychological distress than the holistic view of mental health proposed by the WHO (2014). On the other hand, non‐specific psychological distress is a common symptom in a broad range of mental disorders (Kessler et al., 2002) and all three instruments used in this study have evidence suggesting they can function as valid screening tools in the general population (Gill et al., 2007; Rumpf et al., 2001). Hence, while the current study might not necessarily reflect mental health in its entirety, it does capture important aspects of mental health.

Knowing the crude age pattern of mental health (or psychological distress) is highly important, as it indicates which age groups are at risk for mental health problems. A logical further step then is to see which variables drive this age pattern, so that perhaps specific policies and interventions can be targeted at improving the mental health of at‐risk groups. To identify potential variables, future studies could investigate which variables correlate with the age pattern of mental health, after which researchers can focus on identifying causality. Both of these steps are outside the scope of the current study.

From our background section we conclude that there is no panacea when it comes to APC estimation. Any method for identifying APC effects either has a large probability of misspecification or is underidentified. As a result, while we can derive an unbiased estimate for the second derivative, when estimating the first derivative different methods will lead to different biases and different outcomes. Consequently, any study reporting results from an APC analysis should be interpreted with caution. Our results indeed show that using different methods that require different assumptions about period or cohort effects, can produce different results. Our first approach for estimating the linear age‐effect required the assumption that there is no linear period trend. The results from this estimation implied that mental health is inversely U‐shaped over an individual's life course, where individuals reach their best mental health at age 85 and 44 for women and men, respectively. Our second approach, using varying cohort restrictions, shows much more uncertainty about the linear age‐effect, indicating that while mental health might be inversely U‐shaped over the life course, it could also be continuously increasing or decreasing over age. Hence, only presenting the estimates based on the assumption of no linear period trend would not have appropriately reflected the uncertainty surrounding the estimate of the linear age‐effect.

However, we can be certain that our estimate of the second derivative is unbiased. Hence, this study indicates that there is likely no U‐shape in mental health over age and tentative evidence that mental health could follow an inverse U‐shape over the life course.

With regard to policy, additional research is necessary to determine the conclusive age profile and the determinants thereof. Nevertheless, policy cannot wait until all remaining uncertainty has been resolved before designing and implementing interventions in the here and now. Our analyses suggest that mental health is either inversely U‐shaped, continuously increasing, or continuously decreasing over age. Consequently, young and older adults (as opposed to, e.g., the middle aged) appear to be potentially vulnerable groups. Although our analyses do not provide certainty on which of the two groups is (most) vulnerable, and their vulnerability might also differ by gender, our results indicate that the middle aged are, on average, not amongst the potentially most vulnerable. Therefore, with regard to that contemporaneous need for policy, our analysis suggests the importance of aiming policy at young as well as older adults.

## 8 CONCLUSION

This study investigated how mental health changes over the life‐cycle by first employing an unbiased estimator for the second derivative of the age pattern, after which the linear age‐effect was estimated by widely varying the restrictions on cohorts to reach identification. While a significant body of literature suggests that the age‐profile of mental health might be U‐shaped, we find no evidence for a U‐shape. On the contrary, our results indicate that the relationship between mental health and age could potentially follow an inverse U‐shape, where individuals experience a mental health high at some point during their lives. This finding is highly societally relevant as it suggests that the young and older adults might be particularly at risk for mental health problems. These findings may inform prevention and treatment policies aimed at ameliorating the significant individual and societal costs associated with mental health problems.

## AUTHOR CONTRIBUTIONS

**Hermien Dijk**: Conceptualization; Methodology; Formal analysis; Writing – Original Draft; Visualization. **Jochen Mierau**: Conceptualization; Writing – Review & Editing; Supervision; Funding Acquisition.

## CONFLICT OF INTEREST

This research was financed by Accare. Apart from the funding, Accare was not involved in the study design, the collection, analysis and interpretation of the data, the writing of the article, or in the decision to submit the research for publication. Both authors have no further competing interests to declare.

## ETHICS STATEMENT

No new data was created in this study. Hence, ethical approval was not required.

## Supporting information

Supporting Information S1

Supporting Information S2

## Data Availability

Data sharing is not applicable to this article as no new data were created or analyzed in this study.

## ENDNOTES

^1^
Assuming cohort effects are not decreasing over time for a population with constant or increasing cohort sizes over time, only limited decreases in cohort effects over time with increasing cohort sizes, or only limited decreases in cohort sizes and increasing cohort effects over time.

^2^
Cheng et al. (2017) claim that they can identify the linear trend in age (i.e., the first derivative). See Section 3.1.1 for further discussion.

^3^
Using this method they effectively estimate the second derivative with respect to age of a life‐satisfaction/well‐being equation.

^4^
Conversely, if the second derivative of age indicates the existence of a U‐shape, but the first derivative is sufficiently small, mental health, life satisfaction, or well‐being is nevertheless continuously decreasing over the life course.

^5^
Van Landeghem (2012) uses unaltered year dummies, De Ree and Alessie (2011) use Deaton Paxson year dummies (Deaton & Paxson, 1994). Both methods give equivalent estimates for the second derivative in our case.

^6^
Since *age*_*i*,*t*_ = *age*_*i*,*t*−1_ + 1.

^7^
Or one fewer year dummy and a constant.

^8^
For more details see the working paper version of this paper (Dijk & Mierau, 2019)

^9^
A Stata ado‐file that applies this procedure in the single command **ageff** can be found in the supplementary material.

^10^
Using the untransformed data does not alter the results.

^11^
When split by gender the summary statistics show no substantial differences between men and women, see Tables A1 and A2 in Appendix A1.

^12^
Age groups within each cohort that consisted of less than 100 individuals were left out of the graph as the small samples resulted in volatile K6 scores for those age groups.

^13^
Although the figures also depict the cluster robust 95% confidence intervals of each separate estimate, it should be noted that these confidence intervals do not account for the fact that we have done over 70 estimations of the same coefficient.

^14^
We did not use cohort restrictions on cohorts born before 1920 and after 1993, since these cohorts contain too few individuals (less than 100) to provide accurate results.

^15^
When estimating age‐patterns, adding control variables other than those controlling for cohort or period effects is potentially troublesome, since, by definition, aging is an exogenous process that cannot be affected by commonly included control variables, such as income and marital status (Bittmann, 2020; Easterlin, 2006). In fact, it is more likely that these control variables are in some manner a result of the aging process, which would lead to a bad control problem (Angrist & Pischke, 2009; Glenn, 2009). Moreover, some of the included control variables might not just be endogenous in the sense that they are affected by age (e.g., at certain ages individuals are more likely to have children), but they might also be bad controls in the sense that the control variables might be influenced by an individual's mental health (Bartram, 2020; Glenn, 2009). For example, individuals that are satisfied with their life might be more likely to marry (Glenn, 2009).

^16^
Using first differences of the control variables in our estimation of the second derivative implies the inclusion of level control variables in Equation (1).

^17^
The LISS panel is a representative sample of Dutch individuals who participate in monthly Internet surveys. The panel is based on a true probability sample of households drawn from the population register. Households that could not otherwise participate are provided with a computer and Internet connection. A longitudinal survey is fielded in the panel every year, covering a large variety of domains including work, education, income, housing, time use, political views, values and personality.

^18^
2002; 2004; 2006; 2008; 2010; 2012; 2014; 2016.

^19^
Pooled OLS with clustered standard errors at the individual level on our data gives estimates for *β*_2_ that are closer to zero (*β*_2_ = −0.002, *p* < 0.01, no cohort controls) or statistically insignificant (*β*_2_ = −0.001, *p* > 0.10, using 10‐year cohort dummies) (detailed estimation results available on request) compared to the estimate found in our second‐derivative estimation (*β*_2_ = −0.004, *p* < 0.01). Hence, while assuming cohort effects are zero, or using superfluous parameter restrictions, did not change the sign of our estimate, it does seem to result in an upward bias.

## APPENDIX A GRAPHS AND FIGURES

### A1 Summary statistics

**TABLE A1:** Summary statistics: women

	*N*	Mean	Standard deviation	Minimum	Maximum
Age	7856	38.59	15.97	16	98
Year of birth	7856	1965.97	18.13	1903	1997
Year	7856	2004.56	4.73	2001	2015
K‐6 score (0–100)	7856	83.17	17.40	0	100

*Note*: Summary statistic at first observation (baseline) for each individual.

**TABLE A2:** Summary statistics: men

	*N*	Mean	Standard deviation	Minimum	Maximum
Age	6395	39.43	15.61	16	99
Year of birth	6395	1966.25	17.66	1902	1997
Year	6395	2005.68	5.06	2001	2015
K‐6 score (0–100)	6395	84.86	17.06	0	100

*Note*: Summary statistic at first observation (baseline) for each individual.

**TABLE A3:** Population summary statistics: individuals with at least two consecutive scores

	*N*	Mean	Standard deviation	Minimum	Maximum
Age	11,124	38.67	15.51	16	96
Year of birth	11,124	1965.24	17.47	1905	1995
Year	11,124	2003.90	4.08	2001	2013
Gender					
Female	6733	60.53%			
Male	4391	39.47%			
K‐6 score (0–100)	11,124	84.53	16.69	0	100

*Note*: Summary statistic at first observation (baseline) for each individual.

### A.3 Attrition

**TABLE A4:** Estimation results: second derivative

ΔK6 (0–100)	Individuals present in all waves
2*age*_*i*,*t*_	−0.003**
	(0.002)
Constant	0.586***
	(0.215)
Clustered SE	Yes
Observations	19,060
Number of ID	3812

*Note*: Clustered standard errors in parentheses.

****p* < 0.01, ***p* < 0.05, **p* < 0.1.

### A.4 Control variables

**TABLE A5:** Estimation results: second derivative with controls

ΔK6 (0–100)	Full sample	Women	Men
2*age*_*i*,*t*_	−0.003**	−0.003*	−0.003*
	(0.001)	(0.002)	(0.002)
Constant	0.519***	0.653***	0.341
	(0.169)	(0.224)	(0.263)
Clustered SE	Yes	Yes	Yes
Observations	36,016	22,271	13,745
Number of ID	10,915	6623	4292

*Note*: Clustered standard errors in parentheses. Estimations include controls for individual labor income (dummies indicating the income decile), marital status, education level dummies, employment status dummies, state dummies and a dummy variable denoting whether the household included children under 18. Sample sizes differ from the main analyses due to missing values in the control variables.

****p* < 0.01, ***p* < 0.05, **p* < 0.1.

### A.5 Netherlands and Germany

**TABLE A6:** Estimation results: second derivative

	Netherlands	Germany
2*age*_*i*,*t*_	0.001	−0.006***
	(0.002)	(0.001)
Constant	0.425	0.800***
	(0.267)	(0.137)
Clustered SE	Yes	Yes
Sample weights		Yes
Observations	33,593	111,594
Number of ID	9285	35,246

*Note*: Cluster robust standard errors in parentheses.

****p* < 0.01, ***p* < 0.05, **p* < 0.1.
